# EGFR-specific CAR-T cells trigger cell lysis in EGFR-positive TNBC

**DOI:** 10.18632/aging.102510

**Published:** 2019-12-04

**Authors:** Yan Liu, Yehui Zhou, Kuo-Hsiang Huang, Ying Li, Xujie Fang, Li An, Feifei Wang, Qingfei Chen, Yunchao Zhang, Aihua Shi, Shuang Yu, Jingzhong Zhang

**Affiliations:** 1 The Key Laboratory of Bio-Medical Diagnostics, Suzhou Institute of Biomedical Engineering and Technology, Chinese Academy of Sciences, Suzhou 215163, P. R. China; 2 Changchun Institute of Optics, Fine Mechanics and Physics, Chinese Academy of Sciences, Changchun 130033, P. R. China; 3 The First Affiliated Hospital of Soochow University, Soochow University, Suzhou 215006, P. R. China; 4 Xuzhou Medical University, Xuzhou 221004, P. R. China; 5 Tianjin Guokeyigong Science and Technology Development Company Limited, Tianjin 300399, P. R. China; 6 Zhengzhou Institute of Engineering and Technology Affiliated with SIBET, Zhengzhou 450001, P. R. China

**Keywords:** triple-negative breast cancer, human epidermal growth factor receptor, chimeric antigen receptor engineered T cells

## Abstract

Triple-negative breast cancer (TNBC) is an aggressive cancer subtype for which effective therapies are lacking. Epidermal growth factor receptor (EGFR) is overexpressed in various types of TNBC cells, and several EGFR-specific immunotherapies have been used to treat cancer patients. Chimeric antigen receptor engineered T (CAR-T) cells have also been used as cancer therapies. In this study, we generated two types of EGFR-specific CAR-modified T cells using lentiviral vectors with DNA sequences encoding the scFv regions of two anti-EGFR antibodies. The cytotoxic and antitumor effects of these CAR-modified T cells were examined in cytokine release and cytotoxicity assays *in vitro* and in tumor growth assays in TNBC cell line- and patient-derived xenograft mouse models. Both types of EGFR-specific CAR-T cells were activated by high-EGFR-expressing TNBC cells and specifically triggered TNBC cell lysis *in vitro*. Additionally, the CAR-T cells inhibited growth of cell-line- and patient-derived xenograft TNBC tumors in mice. These results suggest that EGFR-specific CAR-T cells might be a promising therapeutic strategy in patients with high-EGFR-expressing TNBC.

## INTRODUCTION

Triple-negative breast cancer (TNBC) cells, which lack estrogen receptor (ER), progesterone receptor (PR) and human epidermal growth factor receptor 2 (HER2), grow faster than most other breast cancer cell types. TNBC accounts for 15~20% of all breast cancer diagnoses [1]. Because TNBC cells lack the targets upon which specific immunotherapies and hormone therapies act, TNBC patients can only receive non-specific treatments such as chemotherapy and radiotherapy after surgery [2]. In addition, the cytotoxicity of these treatments usually leads to severe adverse effects such as pancytopenia, nausea, and diarrhea [1]. Furthermore, recurrence rates are higher for TNBC patients treated with these therapies than for other breast cancer patients [3]. Effective therapeutic strategies with specific targets are therefore needed to improve treatment efficacy in TNBC.

Of the several biomarkers that are abnormally expressed in TNBC, mutations and copy number alterations have received the most attention. BRCA1/2 is the predominant mutation in TNBC and is present in approximately 73% of TNBC patients [4]; PARP inhibitors or platinum therapy are used to treat these patients. TP53 and PIK3CA also have high mutation rates of about 50–80% and 10–20%, respectively, in TNBC [5]. In addition, aberrant expression of NOTCH family members and MEK kinase have been described in TNBC. Different inhibitors, such as PI3-K inhibitors, MEK inhibitors, and γ-secretase inhibitors, have been used to treat these molecular subtypes of TNBC [5–7]. However, most of the proteins that are abnormally expressed in TNBC are intracellular proteins rather than membrane proteins, which prevents their use as direct targets for TNBC treatments.

EGFR (HER1), a member of the EGFR family of tyrosine kinases, plays important roles in TNBC progression [8]. Activation of EGFR by ligand binding induces either homodimerization or heterodimerization of EGFR receptors, which leads to autophosphorylation of their tyrosine kinase domains [9, 10]. This autophosphorylation recruits a series of downstream signaling pathways such as PI3K/AKT and Ras/Raf/ MEK/ERK. An EGFR-associated gene expression profiling study demonstrated that overexpression of EGFR was found in 45-70% of TNBC patients and was associated with poorer prognosis [11]. However, EGFR mutations are rare in Chinese, Japanese, Korean, European, Australian, and American TNBC patients [12–21]. A previous study identified EGFR gene mutations in exons 19 and 21, which encode parts of the tyrosine kinase domain, but not in exons that encode the extracellular domains [12]. The extracellular domain of EGFR may therefore be an ideal tumor-specific epitope for TNBC therapies [22]. Several anti-EGFR monoclonal antibodies (such as Cetuximab and Panitumumab) and small molecule tyrosine kinase inhibitor (TKIs) (such as Gefitinib and Neratinib) have been tested in TNBC clinical trials. However, many of the patients in those trials responded poorly or developed resistance to these molecules [8, 22, 23]. Novel treatments that target EGFR in TNBC patients are therefore needed.

Chimeric antigen receptor-engineered T cell (CAR-T) therapy has emerged as a promising immunotherapeutic strategy in cancer treatment [24]. Chimeric antigen receptors are recombinant T-cell receptor proteins comprised of extracellular antigen-binding domains, transmembrane domains, and intracellular signaling domains [25, 26]. Various CARs can be created by fusing variable single-chain fragments from a specific anti-tumor monoclonal antibody with at least one intracellular domain from a T-cell receptor [27–30]. The first CARs expressed in T cells were genetically engineered by recombining extracellular domains with transmembrane domains and T-cell receptor CD3ζ chains. However, T cells expressing these CARs were unable to maintain long-term adaptive immunity because they could not mediate other co-stimulatory signals. Recently, 3^rd^ generation CARs have been designed that contain an extracellular binding domain, a hinge region, a transmembrane domain, and an intracellular domain. The extracellular binding domain contains a single-chain variable fragment (scFv) derived from a tumor antigen-reactive antibody. The specific hinge and transmembrane domains are usually connected to extracellular binding and intracellular signaling domains, respectively. The intracellular domain includes both a signaling domain (CD3ζ) for mediating T cell activation and co-stimulatory domains (CD28 and 4-1BB) for enhancing T cell functions such as proliferation, resistance to apoptosis, cytokine secretion, and persistence. These 3^rd^ generation antigen-specific CAR-T cells inhibited tumor cell growth much more efficiently than first generation cells [31–34]. Furthermore, CAR-T treatments have yielded encouraging results in a variety of tumors, such as breast cancer [35, 36], lung cancer [37], colorectal cancer [38], malignant pleural mesothelioma [39, 40], neuroblastoma [41, 42], and pancreatic cancer [43, 44], in preclinical or clinical trials.

In this study, we generated CAR-T cells to specifically target EGFR in TNBC cells by fusing an anti-EGFR single-chain variable fragment (scFv) from a novel anti-EGFR antibody with an artificially combined receptor molecule and examined their antitumor effects.

## RESULTS

### EGFR expression in three TNBC cell lines

**Figure 1:**
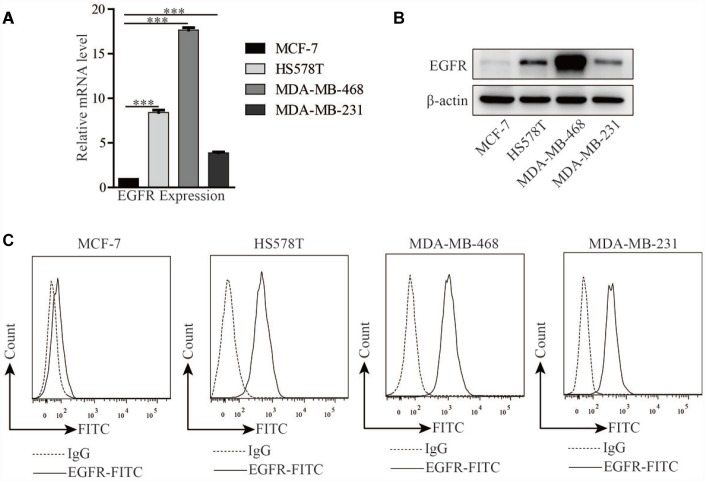
**EGFR expression in breast cancer cell lines.** EGFR expression in TNBC cell lines (HS578T, MDA-MB-468, and MDA-MB-231) and MCF-7 cells detected by (**A**) real-time RT PCR, (**B**) Western blot, and (**C**) flow cytometry. Error bars represent means ± SEM. T-tests were used for statistical analysis; ***p < 0.001.

Real-time RT-PCR and Western blotting were used to examine EGFR RNA transcript and protein levels in the HS578T, MDA-MB-468, MDA-MB-231, and MCF-7 breast cancer cell lines. EGFR mRNA levels were about 3-17 times higher in the TNBC cell lines (HS578T, MDA-MB-468, and MDA-MB-231) than in the non-TNBC MCF-7 cell line (Figure 1A). Similarly, EGFR protein levels were higher in the TNBC cell lines than in the MCF-7 cell line (Figure 1B). Additionally, phosphorylated EGFR levels with or without serum depletion were similar to those of EGFR in all four cell lines (Supplementary Figure 2). Flow cytometry analysis using an anti-EGFR antibody confirmed that the amounts of EGFR protein expressed on cell membranes were correlated with total EGFR transcript levels in all four cell lines (Figure 1C). Based on these results, these four cell lines were used to investigate the antitumor activity of EGFR-specific CAR-modified T cells in subsequent experiments.

### Generation and characterization of EGFR-specific CAR-T cells

**Figure 2:**
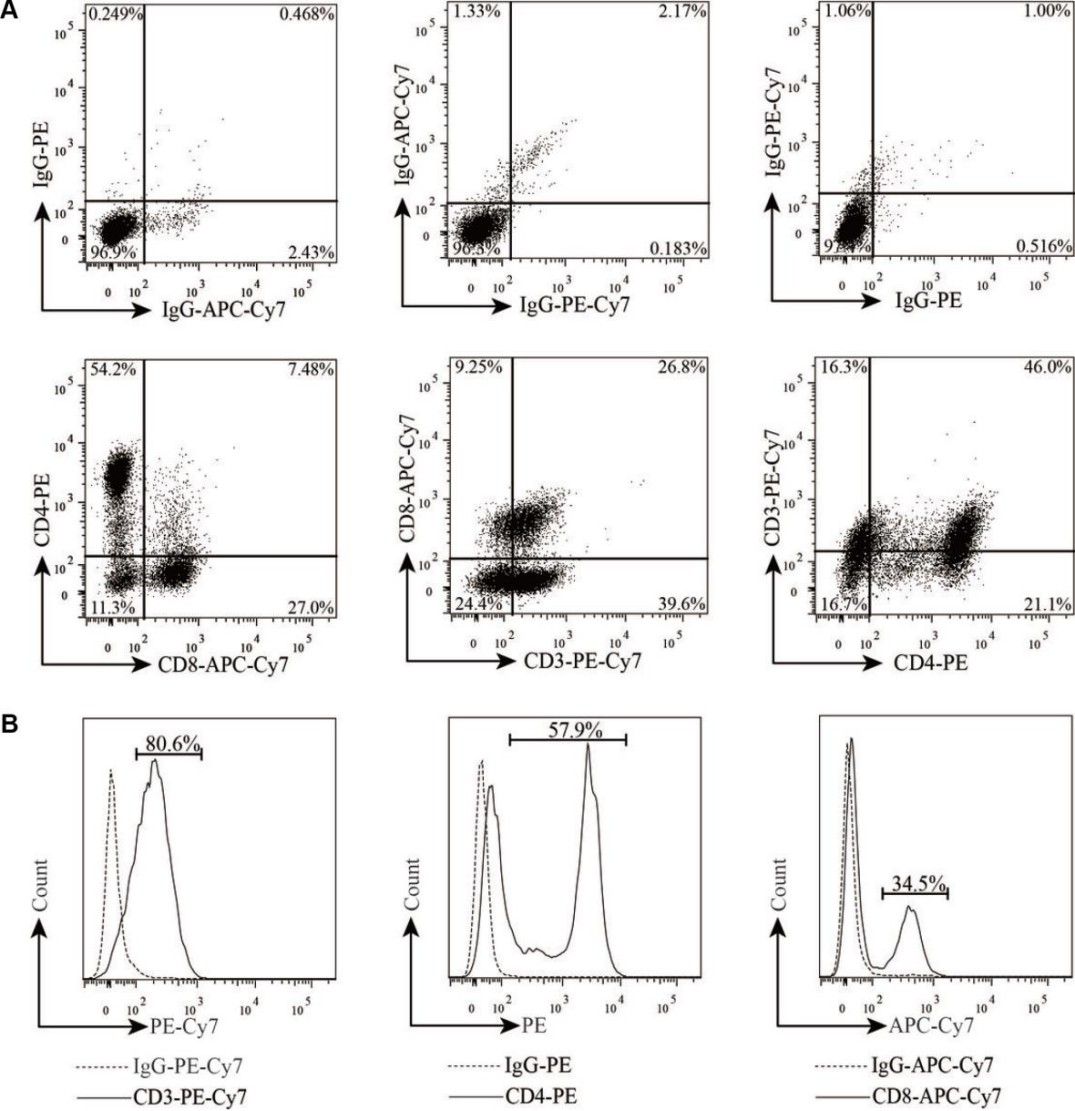
**Characterization of T lymphocytes from PBMCs.** (**A**–**B**) T cell phenotypes and subsets were examined by flow cytometry after labeling with anti-CD3-PE-Cy7, anti-CD4-PE, and anti-CD8-APC-Cy7.

**Figure 3:**
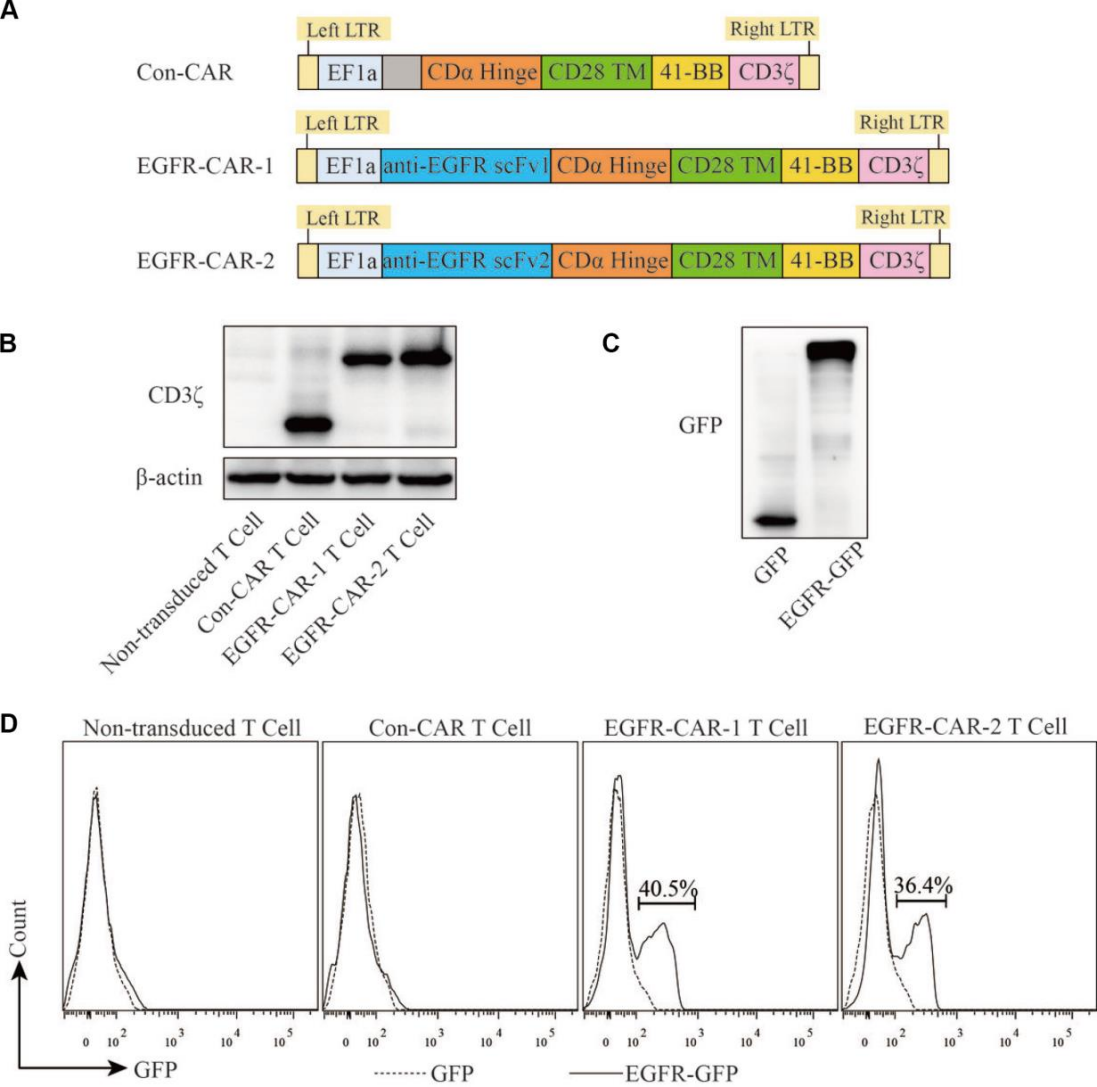
**Generation, isolation, and characterization of EGFR-specific CAR T lymphocytes.** (**A**) Schematic illustration of Con-CAR, EGFR-CAR-1, and EGFR-CAR-2. (**B**) Expression of exogenous CD3ζin non-transduced T cells, con-CAR T cells, EGFR-CAR-1 T cells, and EGFR-CAR-2 T cells was measured using Western blots; β-actin was used as an endogenous control. (**C**) GFP and EGFR-GFP antigens were detected by Western blot. (**D**) Transduced T cells were stained with GFP and EGFR-GFP antigen and then detected by flow cytometry.

To generate EGFR-specific CAR-T cells, human primary T cells were activated with IL-2, isolated from PBMCs cultures using anti-CD3/CD28 beads, and further characterized using flow cytometry analysis with anti-CD3, CD4, and CD8 antibodies. After 10 days of culture, the isolated cell population contained high percentages of potential T cells that were CD3-positive (~61–85%), CD4-positive (~28–58%), and CD8-positive (~19%–48%) (Figure 2A and 2B). These potential T cell populations were then treated with lentiviral vectors that carried one of two EGFR-specific CARs (EGFR-CAR-1 and EGFR-CAR-2) or control CAR (Con-CAR). (Figure 3A). To determine whether EGFR-specific or control CAR-T cells were generated, Western blot analysis using anti-CD3ζ antibody was performed to confirm the expression of CARs in transduced T cells (Figure 3B). Non-transduced and transduced T cells were then treated with purified EGFR-GFP or GFP protein and analyzed by flow cytometry to determine whether EGFR-specific CAR-T cells were able to recognize EGFR *in vitro* (Figure 3C and 3D). Approximately 40% of the EGFR-CAR-1 or EGFR-CAR-2 T cells were labeled with EGFR-GFP but not GFP (Figure 3D), indicating that EGFR-specific CAR-T cells were successfully generated.

### EGFR-specific CAR-T cells trigger TNBC cell lysis *in vitro*

**Figure 4:**
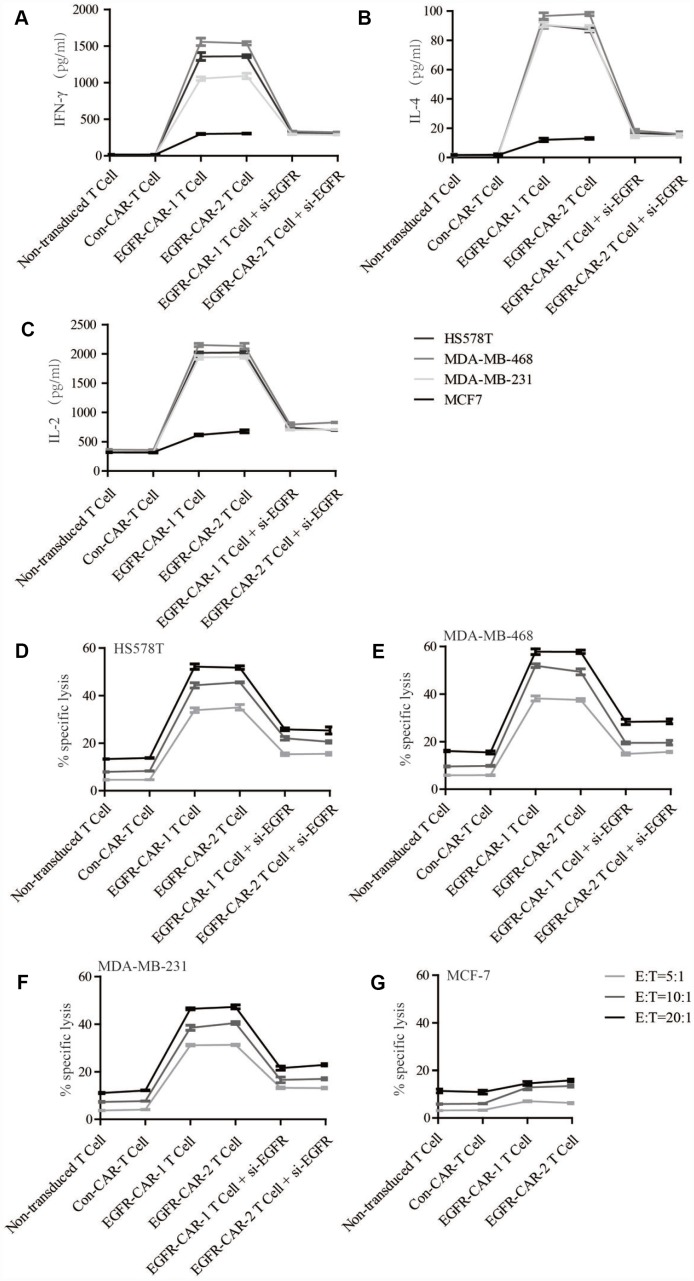
**Cytokine release and cytotoxicity assay.** Cytokine release in target cells in response to effector non-transduced T cells, con-CAR-T cells, EGFR-CAR-1 T cells, and EGFR-CAR-2 T cells. Effector cells were co-cultured with target cells (HS578T, MDA-MB-468, MDA-MB-231, and MCF-7) at an E:T ratio of 10:1 for 24h. (**A**) IFN-γ, (**B**) IL-4, and (**C**) IL-2 levels were assayed in the co-culture supernatants. Cytotoxicity was measured in each group using a standard LDH release assay. Effector cells were co-cultured with (**D**) HS578T, (**E**) MDA-MB-468, (**F**) MDA-MB-231, and (**G**) MCF-7 target cells at E:T ratios of 5:1, 10:1, or 20:1 for 24h.

Next, we examined whether EGFR-specific CAR-T cells expressing either of the two EGFR-CARs were specifically activated upon interaction with TNBC cells (HS578T, MDA-MB-468, MDA-MB-231) expressing high levels of EGFR *in vitro*. Cytokine release assays were performed to quantify relative amounts of Tc1 (IFN-γ and IL-2) and Tc2 (IL-4) cytokines secreted in co-cultured systems containing T cells (transduced or non-transduced) and breast cancer cells (TNBC and non-TNBC). EGFR-specific CAR-T cells co-cultured with TNBC cells secreted significantly higher levels of Tc1 and Tc2 cytokines than those co-cultured with MCF-7 cells (Figure 4A–4C). Additionally, both non-transduced and con-CAR T cells were co-cultured with either TNBC or non-TNBC cells secreted levels of Tc1 and Tc2 cytokines similar to the control (Figure 4A–4C). To determine whether elevated EGFR expression in TNBC cells was correlated with the activation of EGFR-specific CAR-T cells *in vitro*, cytokine release assays were performed to measure cytokine secretion from T cells co-cultured with siRNA-induced EGFR knockdown TNBC cells (Supplementary Figure 3A and 3B). Reduced EGFR expression in TNBC cells was associated with lower cytokine secretion from EGFR-specific CAR-T cells (Figure 4A–4C). These data suggest that activation of EGFR-specific CAR-T cells *in vitro* is likely a result of increased EGFR expression in TNBC cells (Supplementary Table 1).

**Figure 5:**
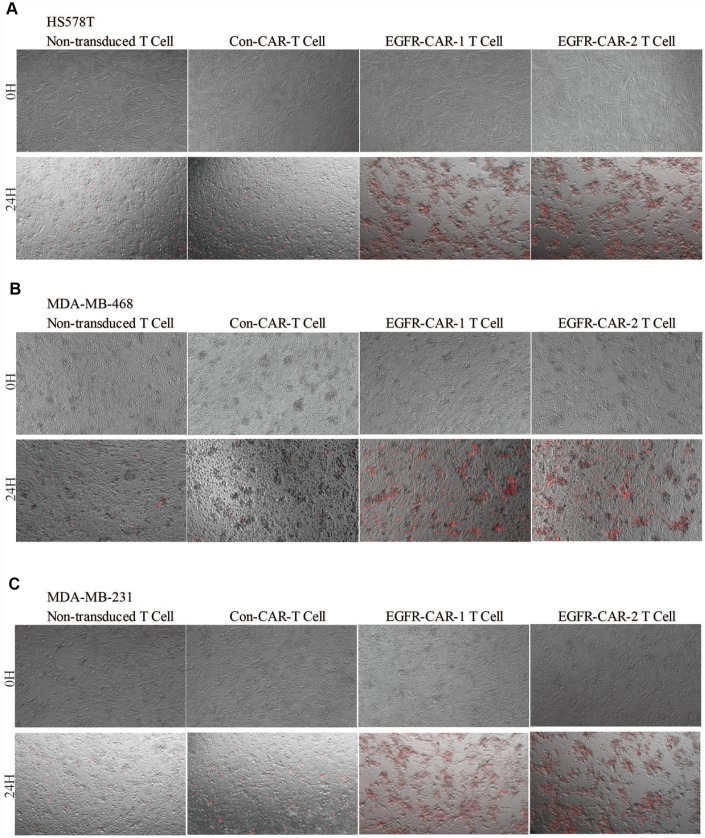
**TNBC cell lysis assay.** (**A**) HS578T, (**B**) MDA-MB-468, and (**C**) MDA-MB-231 cells were labeled with YOYO-3 (red). Non-transduced T cells, con-CAR-T cells, EGFR-CAR-1 T cells, and EGFR-CAR-2 T effector cells were co-cultured with target cells at an E:T ratio of 10:1 for 24h.

Next, we investigated whether activated EGFR-specific CAR-T cells were able to specifically trigger cell death in TNBC cells. TNBC-specific lysis percentage was examined in a cytotoxicity assay that measured ratios of LDH activity between effector T cells and target breast cancer cells (E/T ratio) in the co-cultured systems. As expected, a higher E/T ratio between the EGFR-specific CAR-T cells and the high-EGFR-expression TNBC cells led to higher specific lysis percentages in the co-cultured systems (Figure 4D–4G). Conversely, a higher E/T ratio between the EGFR-specific CAR-T cells and the low-EGFR-expression MCF-7 cells did not result in an increased specific lysis percentage in that co-cultured system (Figure 4D–4G). In addition, unlike in normal TNBC cells, higher E/T ratios between EGFR-specific CAR-T cells and EGFR-knockdown TNBC cells did not increase specific lysis percentages (Figure 4D–4G and Supplementary Table 1). Furthermore, YOYO™-3 Iodide staining cell lysis assays confirmed that EGFR- specific CAR-T cells triggered much more TNBC cell lysis than con-CAR-T or non-transduced T cell did (Figure 5). Taken together, these results suggest that activated EGFR-specific CAR-T cells likely triggered cell lysis in high-EGFR-expressing TNBC cells *in vitro*.

### Anti-TNBC activity of EGFR-specific CAR-T cells in mouse models

**Figure 6:**
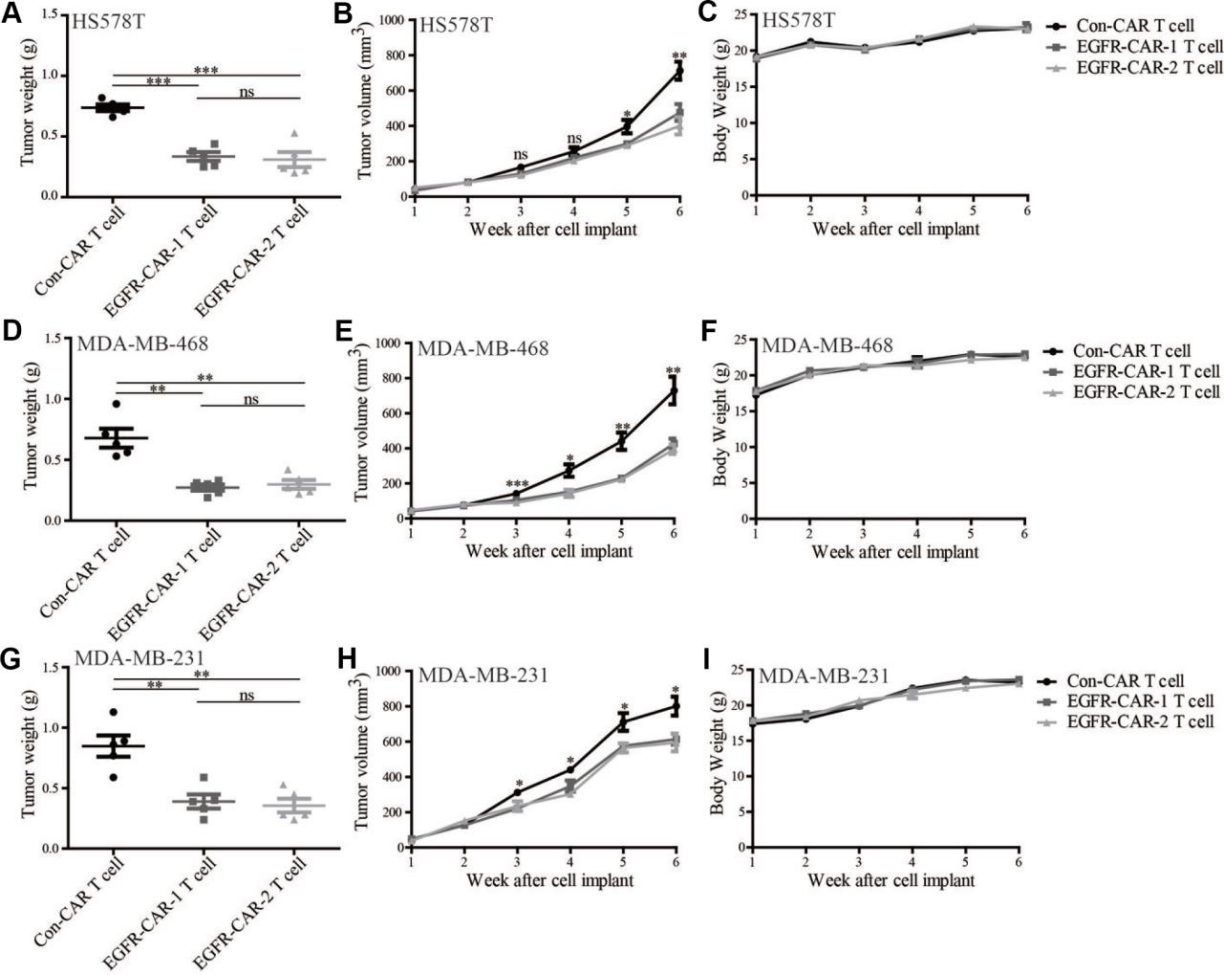
**EGFR-specific CAR-T cells inhibited EGFR-expressing TNBC tumor growth in CLDX mouse model.** Compared to con-CAR-T cells, EGFR-CAR-1 and EGFR-CAR-2 T cells decreased the weights and volumes of tumors induced by (**A**, **B**) HS578, (**D**, **E**) MDA-MB-468, and (**G**, **H**) MDA-MB-231 TNBC cells, but did not affect body weight (**C**, **F**, **I**). Error bars represent means ± SEM. T-tests were used for statistical analysis; *p < 0.05, ** *p* < 0.01, ***p < 0.001.

**Figure 7:**
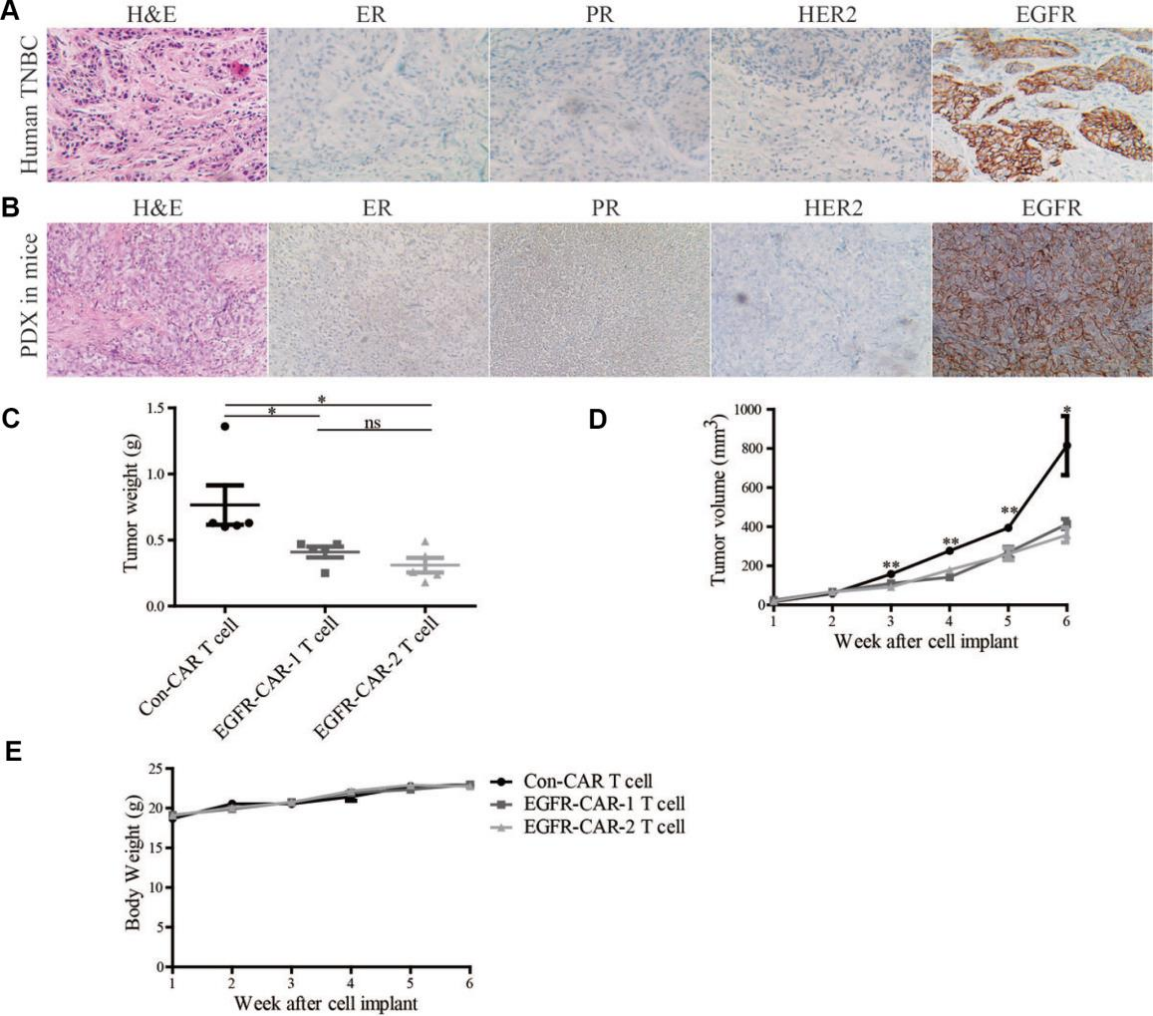
**EGFR-CAR-T cells inhibited high-EGFR-expressing TNBC tumor growth in PDX mouse model.** ER, PR, HER2, and EGFR expression in (**A**) clinical breast cancer samples and (**B**) breast cancer tumors in PDX mice were assessed in immunohistochemical assays. Compared to con-CAR-T cells, EGFR-CAR-1 and EGFR-CAR-2 T cells decreased breast cancer (**C**) tumor weights and (**D**) tumor volumes but did not affect (**E**) body weights. Error bars represent means ± SEM. T-tests were used for statistical analysis; *p < 0.05, **p < 0.01.

To assess whether EGFR-specific CAR-T cells inhibited growth of cell-line-derived TNBC tumors, tumor growth assays were performed after inoculating TNBC cell lines into the mammary fat pads of mice that were injected with either EGFR-specific CAR-T or con-CAR-T cells. The average weights and volumes of xenograft TNBC tumors treated with either of the two EGFR-specific CAR-T cells were lower than those treated with con-CAR-T cells (Figure 6). To further investigate whether EGFR-specific CAR-T cells inhibited growth of patient-derived xenograft (PDX) tumors, tumor growth assays were performed on mice inoculated with cells from TNBC patients. As was the case for cell line-derived tumors, high-EGFR-expressing PDX tumors treated with either of the two EGFR-specific CAR-T cells were smaller than those treated with the con-CAR-T cells (Figure 7A–7D). In addition, immunohistochemistry analyses showed that the EGFR, ER, PR, and HER2 protein expression patterns of the mouse PDX tumors were similar to those of the original TNBC patient tissues (Figure 7A and 7B). Mouse body weights were not affected by treatment with either EGFR-specific CAR-T cells or con-CAR-T cells in either xenograft model (Figure 6C, 6F, 6I, and 7E). Together, these results indicate that EGFR-specific CAR-T cells inhibit the growth of high-EGFR-expressing TNBC tumors in mice.

## DISCUSSION

Antigen-specific CAR-T cells recognize their corresponding antigens via an antigen binding domain. Because activation of CAR-T cells is not required for their interaction with the major histocompatibility complex (MHC) on antigen-presenting cells (APC), tumor cells are unlikely to escape from CAR-T cell immune responses [45]. In this study, we generated two distinct types of third-generation EGFR-specific CAR-T cells. Flow cytometry analyses showed that the EGFR-specific CAR-T cells could specifically recognize EGFR (Figures 2 and 3). Compared to non-transduced T cells and con-CAR-T cells, the EGFR-specific CAR-T cells also had greater cytotoxic effects on high-EGFR-expressing TNBC cell lines (Figures 4 and 5). Furthermore, EGFR-specific CAR-T cells exerted significant anti-tumor effects in both high-EGFR- expressing TNBC xenograft models (Figures 6 and 7). Thus, our research indicates that EGFR-specific CAR-T cells may represent a promising therapeutic strategy against high-EGFR-expressing TNBC.

EGFR plays important roles in cell proliferation, apoptosis, angiogenesis, and other processes related to cancer progression [46–48]. EGFR is highly expressed on TNBC cell membranes [11]. When EGF binds to and activates EGFR, it triggers EGFR homodimerization or heterodimerization and transphosphorylation, which in turn activates downstream molecular signaling [49, 50]. For example, EGFR and JAK bind to and activate STAT via the Src homology 2 domain; STAT then homo- and heterodimerizes and is translocated into the nucleus to trigger expression of downstream genes involved in survival and proliferation. EGFR also recruits Ras GTP-binding protein to activate Ras either directly by binding to Grb2 adapter proteins and guanine exchange factor or indirectly by recruiting Shc adaptor to the receptor docking sites; this triggers a kinase cascade that activates Raf, MEK, and ERK and ultimately phosphorylates transcription factors involved in cell proliferation [48]. In the present study, cell proliferation assays showed that ectopic overexpression of EGFR in the MCF7 cell line, which typically has low EGFR expression, promoted proliferation. Furthermore, EGFR knockdown in the MDA-MB-468 cell line, which normally expresses high levels of EGFR, inhibited cell proliferation (Supplementary Figure 4). However, active ERK levels were not correlated with nuclear EGFR expression in MCF7 or MDA-MB-468 cells (Supplementary Figure 5). Several EGFR-specific monoclonal antibodies targeting the extracellular domain and small molecule TKIs targeting the tyrosine kinase domain of EGFR have been used as cancer therapies [22, 23, 51]. However, many TNBC patients in those trials either responded poorly to the treatments or developed drug resistance [22, 23]. With the development of immunotherapy, CAR-T technology has become one of the most promising strategies for treating solid cancers. CAR-T cells promoted MHC-independent cancer cell death by enabling T cells to specifically recognize scFv binding domains of target cell surface antigens. Upon engagement, CAR-T cells formed a non-classical immune synapse which was required for this effect. The resulting antitumor activity was mediated by the Fas/Fas ligand axis, the granzyme/perforin axis, and the release of cytokines that sensitized the cancer stroma [52]. In the present study, EGFR-specific CAR-T cells, which recognized EGFR more efficiently than Con CAR-T cells (Figure 3D), were activated and increased the secretion of IFN-γ, IL-2, and IL-4 upon co-culture with high-EGFR-expressing TNBC cells *in vitro* (Figure 4A–4C and Supplementary Table 1). In addition, both LDH release and YOYO-3 labeling assays indicated that activated CAR-T cell-induced cytotoxicity was higher in high-EGFR-expressing TNBC cells than in MCF-7 cells *in vitro* (Figures 4 and 5). An LDH release assay also revealed that the cytotoxic effects of EGFR-specific CAR-T cells increased when an activator was used to promote EGFR dimerization as compared to untreated breast cancer cells (Supplementary Figure 6). These results suggest that the efficiency of cell lysis triggered by EGFR-specific CAR-T cells may be dependent on the amount of EGFR present in breast cancer cells. A similar phenomenon has been observed in CAR-T cells targeting other breast cancer proteins [35, 53].

Traditional immunotherapies developed from first-generation antigen-specific CAR-T cell technology are more effective for treating blood cancers than solid cancers [25, 54]. However, third-generation CAR-T cells that affect more intracellular signaling pathways also show increased antitumor activity compared to first-generation CAR-T cells. Our data also show that EGFR-specific CAR-T cells significantly inhibited high-EGFR-expressing TNBC in CLDX (Figure 6) and PDX mouse models (Figure 7). EGFR-specific CAR-T technology might therefore be an important treatment option for TNBC patients who respond poorly or develop resistance to EGFR-specific monoclonal antibodies and small molecule TKIs. EGFR-specific CAR-T cells could be derived either from the patients’ own T cells or from iPSC cells and then injected either into breast tumors or intravenously to treat EGFR-positive breast cancer patients. This approach carries a very low risk of graft-versus-host disease and enables lipid, protein, and carbohydrate antigens to be targeted by T cells in an MHC-independent fashion [55].

In summary, we confirmed that EGFR-specific CAR-T cells were able to efficiently recognize high-EGFR-expressing TNBC cells. Our results revealed that the two distinct types of activated EGFR-specific CAR-T cells inhibited TNBC tumor growth both *in vitro* and in mouse models by upregulating cytokine secretion and promoting cytotoxicity in TNBC cells.

## MATERIALS AND METHODS

### Cell lines and cell culture

Human breast cancer cell lines MDA-MB-231, MDA-MB-468, HS578T, and MCF-7 were all obtained from American Type Culture Collection (ATCC) and used within generation 20 (P20). All cell lines were cultured in Dulbecco’s modified essential medium (DMEM) (Gibco) supplemented with 10% heat-inactivated fetal bovine (Gibco) and 1% penicillin-streptomycin solution (Gibco) in a humidified incubator with 5% CO2 at 37°C.

### Generation of EGFR-specific CAR-modified T cells

Peripheral blood mononuclear cells (PBMCs) were isolated from whole blood from healthy donors using Ficoll density gradient centrifugation. The cells were incubated with 5% CO2 and saturated humidity at 37°C. T cells were obtained by stimulating PBMCs in AIM V medium (Gibco) with anti-CD3/CD28 beads (Invitrogen) and IL-2 (BioGems) for 10 days. Subsequently, T cells were transduced with lentiviral vectors that carried DNA sequences encoding 3^rd^-generation EGFR-CARs (Lenti-EF1a-scFv-3^rd^-CAR) composed of anti-EGFR-specific scFv (1 or 2) linked to a CDα hinge, a CD28 transmembrane domain, and 41-BB and CD3ζ intracellular signaling domains. The sequences of the lentiviral vector DNA constructs used to generate CARs were confirmed (Supplementary Figure 1). EGFR-specific CAR-modified T cells were collected after stimulation with the appropriate antigens and antibodies.

### Real-time RT PCR

Total RNA was extracted using the RNeasy kit (OMEGA). For real-time PCR, cDNAs were synthesized with the PrimeScript RT reagent kit (TaKaRa) and PCR reactions were performed with SYBR Premix Ex Taq (TaKaRa). The following primer sequences were used for PCR: β-Actin, 5′-AACCCTAA GGCCAACCGTGA-3′ and 5′-GTCTCCGGAGTCCATCACAA-3′; EGFR, 5′-AGTATTGATCGGGAGAGCC-3′ and 5′-CCAGGATAAATTGAATGGCAC-3′; CD3 ζ, 5′-GCCAGAACCAGCTCTATA-3′ and 5′-CCTCCGCCATCTTATCTT-3′.

### siRNA transfection

The following EGFR-targeting and negative control siRNAs were purchased from GenePharma (Suzhou): negative control, sense 5′-UUCUUCGAACGUGUCA CGUTT-3′ and antisense 5′-ACGUGACACGUUCGGAGAATT-3′; si-EGFR, sense 5′-GAAUUAAGAGAAGCAACAUTT-3′ and 5′-AUGUUGCUUCUCUUAAUUCCU-3′. Each siRNA was transfected into breast cancer cells with Lipofectamine RNAi MAX (Invitrogen; Carlsbad, CA).

### Western blotting

Western blotting was performed as described previously [56, 57]. The antibody against CD3ζ was purchased from Abcam. Anti-EGFR and anti-P-EGFR (Tyr1068) antibodies were purchased from Cell Signaling Technology. Anti-β-actin antibody was purchased from Sigma. Anti-rabbit and anti-mouse IgG antibodies were purchased from Santa Cruz Biotechnology.

### Flow cytometry procedure and analysis

Breast cancer and EGFR-specific CAR-T cells were quantitated or isolated by flow cytometry using several fluorescence-conjugated antibodies or EGFR-GFP fusion protein according to the manufacturers’ instructions. Anti-Human CD3 (PE-Cy7), anti-Human CD4 (PE), and anti-Human CD8 (APC-Cy7) antibodies, as well as corresponding mouse IgG controls, were purchased from BioGems. The anti-EGFR antibody was purchased from Cell Signaling Technology. Human TruStain FcX™ blocking solution was purchased from BioLegend. Goat anti-Rabbit IgG was purchased from Abcam. Fluorescence was assessed using a BD™ flow cytometer, and the data were analyzed using FlowJo 7.6.1.

### *In vitro* cytokine release assay

1×10^4^ human breast cancer cells (HS578T, MDA-MB-468, MDA-MB-231, and MCF-7) were co-cultured with the optimized number of EGFR-specific CAR-modified T cells in separate wells of 96-well flat-bottom plates. 24h hours later, the medium was collected from the co-cultured system and analyzed using ELISAs to detect the secretion of IFN-γ, IL-4, and IL-2 (Dakewei).

### Cytotoxicity assay

1×10^4^ human breast cancer cells (HS578T, MDA-MB-468, MDA-MB-231, and MCF-7) were co-cultured with the optimized number of EGFR-specific CAR-modified T cells in separate wells of 96-well flat-bottom plates. Medium containing dying and dead cells was then collected from the wells for further analyses. The LDH Cytotoxicity Assay Kit (Beyotime) and YOYO™-3 Iodide (ThermoFisher) were used to measure the cytotoxic activity of CAR-modified T cells according to the manufacturers’ instructions. Cytotoxic activity was analyzed using an enzyme-labeled instrument and live cell imaging system.

### Tumor growth assays in the cell-line-derived xenograft mouse model

The female nude mice were purchased from Shanghai Sushang Biology Technology and maintained under pathogen-free conditions. On day 1 of the experiment, 5×10^6^ TNBC cells were injected into the mammary fat pads of the mice. 1×10^7^ T cells were then injected into the resulting TNBC tumors on days 14, 21, 28, and 35. Vernier calipers were used to measure tumor width and length weekly, and volume was calculated using the formula 1/2 × (l) × (w)^2^ [l: length; w: width].

### Tumor growth assays in the patient-derived xenograft (PDX) mouse model

This method was approved by the medical ethics committee at the Suzhou Institute of Biomedical Engineering and Technology (A-06). Human TNBC tumor tissues were obtained from the First Affiliated Hospital of Soochow University; letters of authorization were signed by all the patients who provided tissues. Cells from these tissues were grafted onto the mammary fat pad of each mouse as described above. 1×10^7^ T cells were injected into resulting PDX tumors with volumes of at least 4 mm^3^ on days 14, 21, 28, and 35. Tumor sizes were measured as described above.

### Immunohistochemical assay

Primary breast tumors and PDX xenograft tumors were fixed with 4% paraformaldehyde, embedded in paraffin blocks, and then micro-dissected into several thin sections. Sections were then deparaffinized and subjected to antigen retrieval in citric acid buffer (pH 3.5) for 15 minutes. Sections were then incubated in 1% hydrogen peroxidase for 10 minutes followed by incubation with HRP-conjugated antibodies against ER, PR, HER2, or EGFR (Cell Signaling Technology) at 4°C overnight. Staining was performed using the HRP-IHC kit according to the manufacturer’s instructions.

### Ethics approval

PBMCs were obtained from healthy donors and patients after informed consent per protocols approved by Suzhou Institute of Biomedical Engineering and Technology. Mice were housed and treated in accordance with Institutional Animal Care and Use Committee guidelines of Suzhou Institute of Biomedical Engineering and Technology (A-06).

## Supplementary Material

Supplementary Figures

Supplementary Table 1
