# Supplementary material for: EGFR-specific CAR-T cells trigger cell lysis in EGFR-positive TNBC
Source: Aging (Albany NY). 2019 Dec 4;11(23):11054–72. doi: 10.18632/aging.102510 (PMC6932924; doi:10.18632/aging.102510)
Supplement: Supplementary Figures [file aging-11-102510-s002..pdf]

SUPPLEMENTARY FIGURES

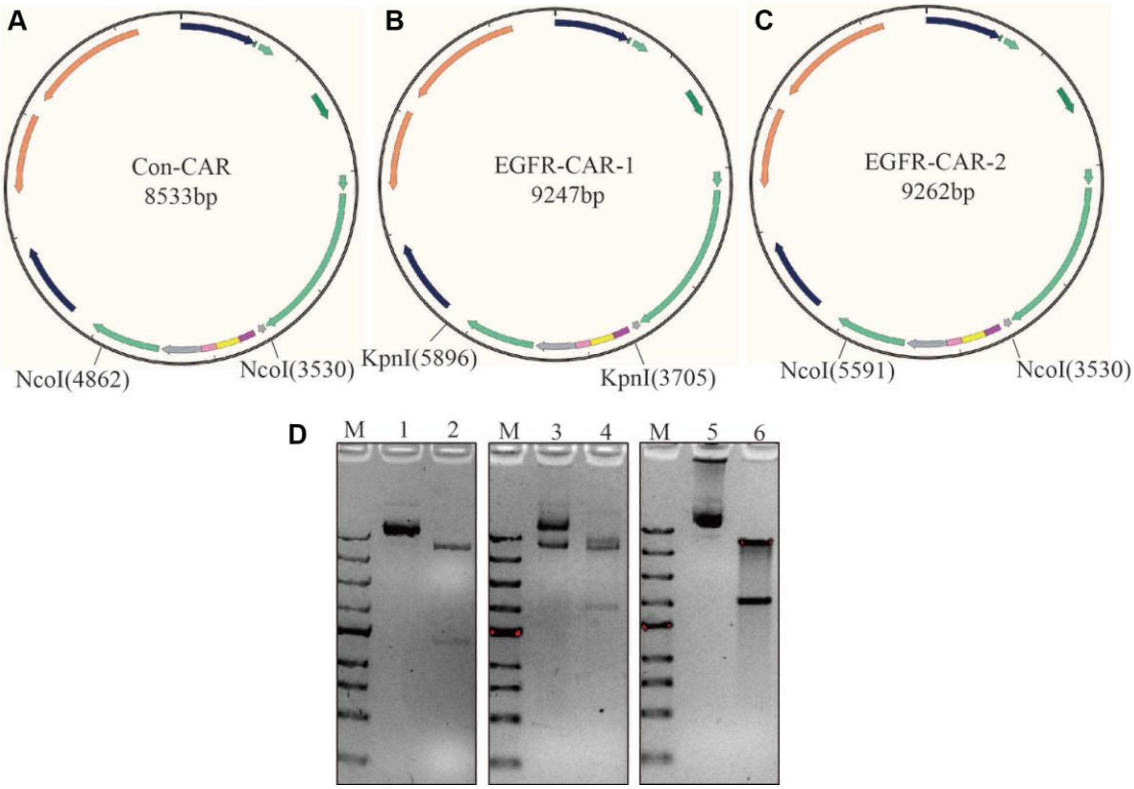

Supplementary Figure 1. Agarose gel electrophoresis of plasmids and restriction-digested DNA products. The structures of (**A**) con-CAR, (**B**) EGFR-CAR-1, and (**C**) EGFR-CAR-2. (**D**) M: 1kb DNA marker; Lane 1: con-CAR plasmid (8533bp); Lane 2: products of restriction digest of con-CAR plasmid using NcoI (7201bp and 1332bp); Lane 3: EGFR-CAR-1 plasmid (9247bp); Lane 4: products of restriction digest of EGFR-CAR-1 plasmid using KpnI (7083bp and 2191bp); Lane 5: EGFR-CAR-2 plasmid (9262bp); Lane 6: products of restriction digest of EGFR-CAR-2 using NcoI (7201bp and 2061bp).

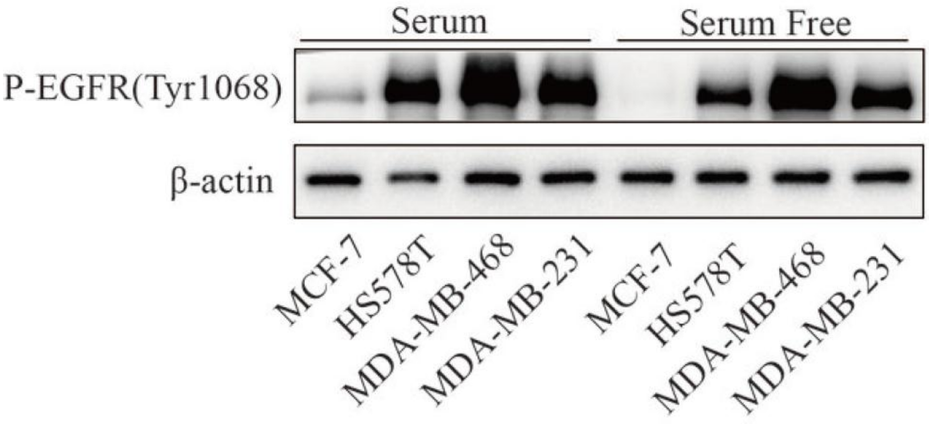

Supplementary Figure 2. P-EGFR expression in breast cancer cell lines. TNBC cell lines (HS578T, MDA-MB-468, and MDA-MB-231) and MCF-7 cells were cultured in 10% FBS or serum free medium for 48h. Phosphorylated EGFR was then detected by Western blot.

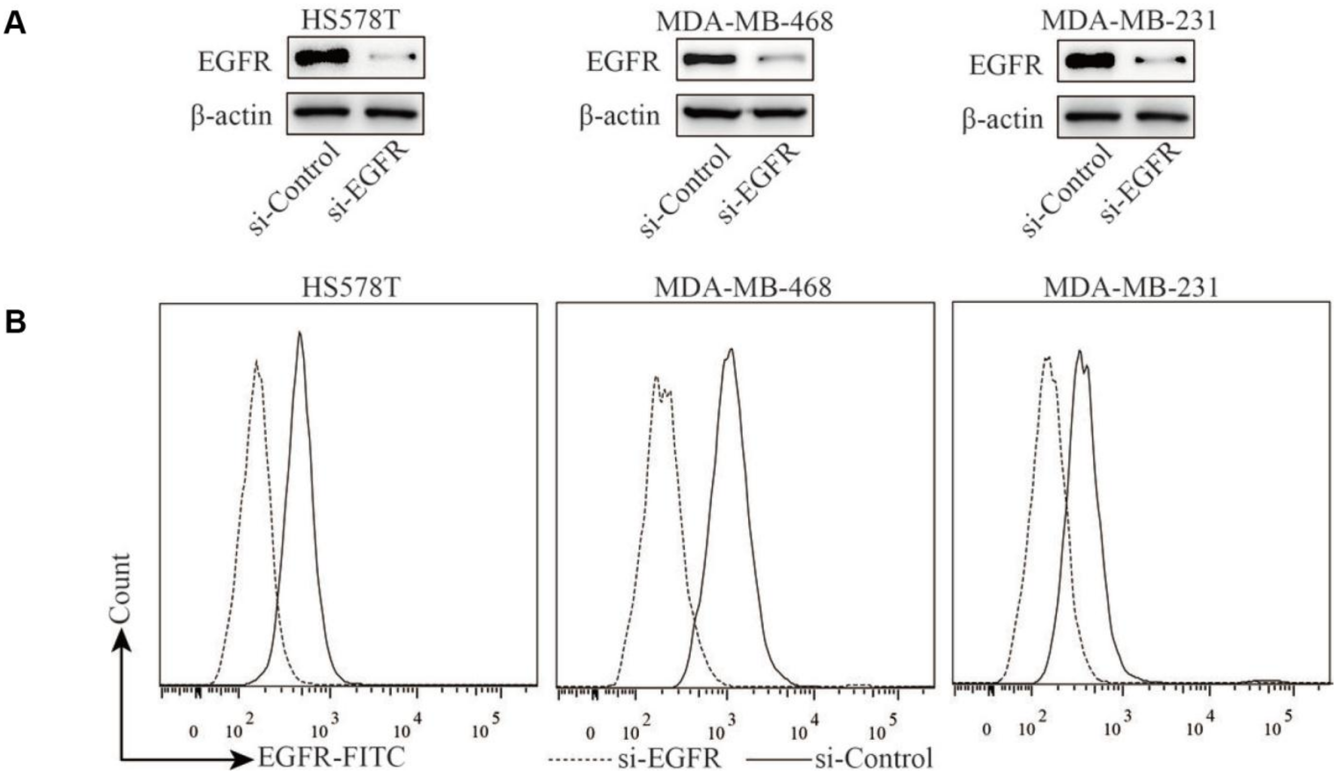

Supplementary Figure 3. Down-regulation of EGFR expression in TNBC cells by siRNA. (**A**) HS578T, MDA-MB-468, and MDA-MB-231 cells were transfected with si-Control or si-EGFR for 48h. EGFR expression was then detected by Western blot. (**B**) EGFR expression detected by flow cytometry.

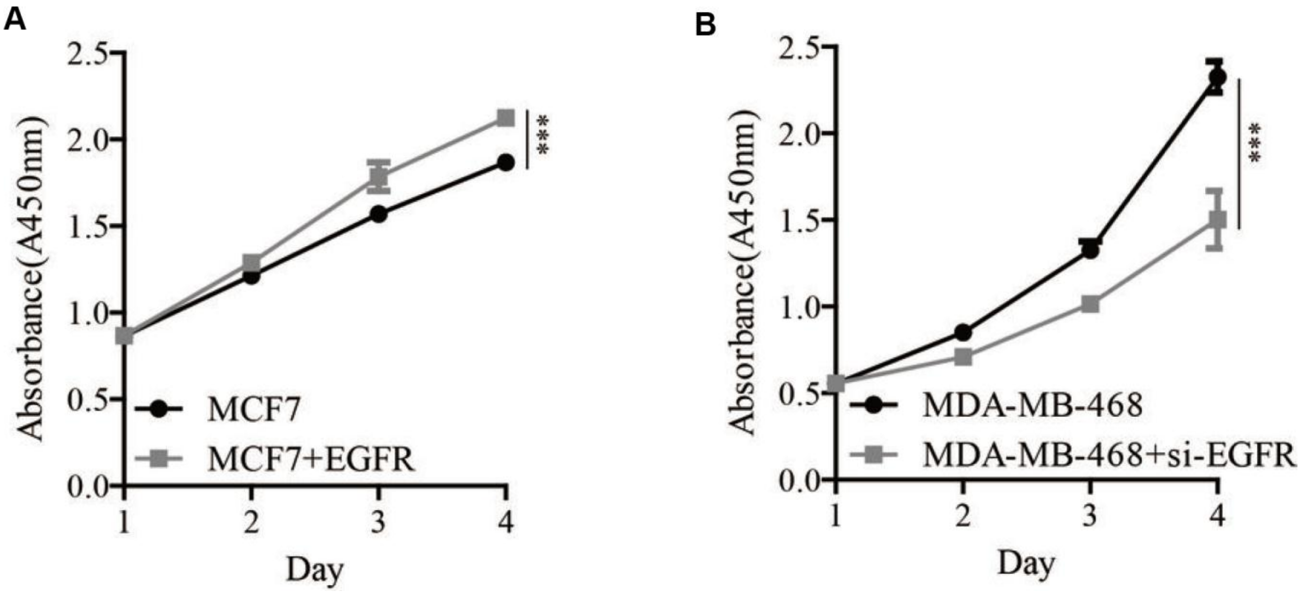

Supplementary Figure 4. EGFR function was detected in breast cancer cell lines with cell proliferation assays. (**A**) Ectopic overexpression of EGFR in MCF7 cells increased proliferation. (**B**) EGFR knockdown in MDA-MB-468 cells decreased proliferation. Error bars represent means ± SEM. T-tests were used for statistical analysis; ***$p < 0.001$.

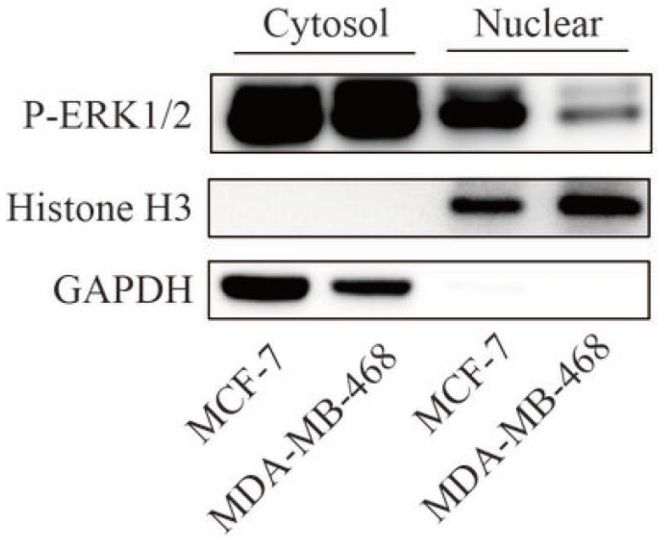

Supplementary Figure 5. Cytosolic and nuclear p-ERK levels in breast cancer cells. Analysis of p-ERK (Thr202/Tyr204) cellular localization by subcellular fractionation. MCF7 and MDA-MB-468 cells were subjected to subcellular fractionation, and Western blot was performed using cytosolic and nuclear fractions. GAPDH and Histone H3 were used as loading controls.

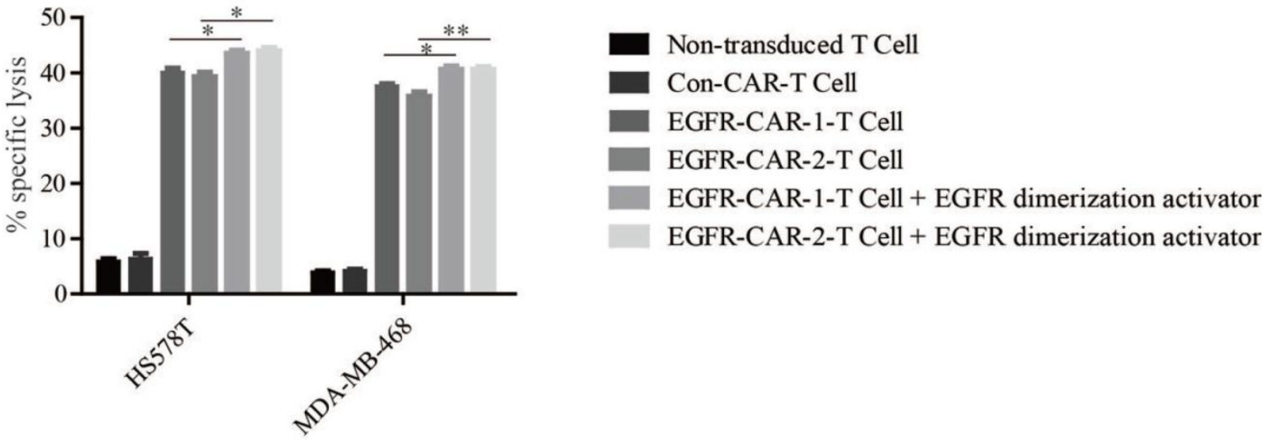

Supplementary Figure 6. Cytotoxicity was measured using a standard LDH release assay. (**A**) HS578T and (**B**) MDA-MB-468 cells were cultured with or without an EGFR dimerization activator (10μM NSC 2281155) for 12h. Effector cells were then co-cultured with target cells at an E:T ratio of 5:1. Error bars represent means ± SEM. T-tests were used for statistical analysis; *$p < 0.05$, **$p < 0.01$.
