# Supplementary material for: EGFR-specific CAR-T cells trigger cell lysis in EGFR-positive TNBC
Source: Aging (Albany NY). 2019 Dec 4;11(23):11054–72. doi: 10.18632/aging.102510 (PMC6932924; doi:10.18632/aging.102510)
Supplement: Supplementary Table 1 [file aging-11-102510-s001..pdf]

SUPPLEMENTARY TABLE

Supplementary Table 1. Cytokine release and cytotoxicity assay data.

**IFN-γ**	**Non-transduced T Cell**	**Con-CAR-T Cell**	**EGFR-CAR-1 T Cell**	**EGFR-CAR-2 T Cell**	**EGFR-CAR-1 T Cell+si-EGFR**	**EGFR-CAR-2 T Cell+si-EGFR**
HS578T	12.7±1.3	17±3	1357.3±94.7	1361±23	315.3±14.7	308±3
MDA-MB-468	19.3±2.7	14.3±1.7	1557±95	1537.3±40.7	335±5	320.3±7.7
MDA-MB-231	18±3	15.3±1.7	1054.3±45.7	1091.7±43.3	294.7±9.3	288±6
MCF7	17.6±1.4	17.7±1.3	299±13	305±10		
**IL-4**	**Non-transduced T Cell**	**Con-CAR-T Cell**	**EGFR-CAR-1 T Cell**	**EGFR-CAR-2 T Cell**	**EGFR-CAR-1 T Cell+si-EGFR**	**EGFR-CAR-2 T Cell+si-EGFR**
HS578T	1.7±0.3	2±1	90.7±4.3	87.3±2.7	16.7±0.3	15.7±3.3
MDA-MB-468	1.7±0.3	1.3±0.7	90.7±3.3	88.3±3.7	14.3±1.7	15±2
MDA-MB-231	1.7±1.3	1.3±0.7	96.7±4.3	98±2	18.3±1.7	16±2
MCF7	1.7±0.3	1.7±1.3	12±2	13±1		
**IL-2**	**Non-transduced T Cell**	**Con-CAR-T Cell**	**EGFR-CAR-1 T Cell**	**EGFR-CAR-2 T Cell**	**EGFR-CAR-1 T Cell+si-EGFR**	**EGFR-CAR-2 T Cell+si-EGFR**
HS578T	316.3±3.7	312.7±9.3	2017.7±27.3	2022.3±62.7	739.7±57.3	696.7±11.3
MDA-MB-468	363.7±6.3	359.7±5.3	2151.7±52.3	2134±64	794.3±34.7	830.3±5.7
MDA-MB-231	319.7±6.3	322.3±5.7	1940±40	1947±31	704.3±10.7	709.7±5.3
MCF7	319.3±4.7	318±3	616.7±23.3	679.3±30.7		
**E:T(HS578T)**	**Non-transduced T Cell**	**Con-CAR-T Cell**	**EGFR-CAR-1 T Cell**	**EGFR-CAR-2 T Cell**	**EGFR-CAR-1 T Cell+si-EGFR**	**EGFR-CAR-2 T Cell+si-EGFR**
5:1	4.62±0.23	4.64±0.04	33.84±1.61	35.03±1.42	15.34±0.9	15.52±0.93
10:1	7.91±0.21	8.31±0.1	44.27±1.85	45.53±0.32	22.03±1.38	20.55±0.68
20:1	13.32±0.12	13.76±0.2	52.12±1.22	51.7±1.42	25.81±0.94	25.31±2.94
**E:T (MDA-MB-468)**	**Non-transduced T Cell**	**Con-CAR-T Cell**	**EGFR-CAR-1 T Cell**	**EGFR-CAR-2 T Cell**	**EGFR-CAR-1 T Cell+si-EGFR**	**EGFR-CAR-2 T Cell+si-EGFR**
5:1	5.88±0.13	5.91±0.3	38.12±1.66	37.52±0.93	14.82±0.95	15.68±0.56
10:1	9.58±0.45	9.82±0.32	51.9±1.22	49.33±1.42	19.44±0.7	19.56±1.89
20:1	16.06±0.35	15.46±0.8	57.78±2.34	57.72±1.45	28.31±1.8	28.48±2.01
**E:T (MDA-MB-231)**	**Non-transduced T Cell**	**Con-CAR-T Cell**	**EGFR-CAR-1 T Cell**	**EGFR-CAR-2 T Cell**	**EGFR-CAR-1 T Cell+si-EGFR**	**EGFR-CAR-2 T Cell+si-EGFR**
5:1	3.74±0.27	4.12±0.1	31.15±0.41	31.3±0.29	13.24±0.45	13.15±0.67
10:1	7.31±0.32	7.69±0.15	38.48±1.07	40.49±0.92	16.57±2.36	17.04±0.57
20:1	11.07±0.38	12.15±0.26	46.4±0.72	47.26±1.56	21.53±1.64	22.95±0.92
**E:T(MCF7)**	**Non-transduced T Cell**	**Con-CAR-T Cell**	**EGFR-CAR-1 T Cell**	**EGFR-CAR-2 T Cell**		
5:1	3.15±0.06	3.26±0.18	7.04±0.41	6.29±0.56		
10:1	5.88±0.24	6.01±0.2	12.85±1.83	13.44±0.67		
20:1	11.37±1.48	10.86±1.58	14.5±1.58	15.82±0.73		
